# A Digital Substance-Use Harm Reduction Intervention for Students in Higher Education (MyUSE): Protocol for Project Development

**DOI:** 10.2196/17829

**Published:** 2020-08-27

**Authors:** Samantha Dick, Vasilis S Vasiliou, Martin P Davoren, Samantha Dockray, Ciara Heavin, Conor Linehan, Michael Byrne

**Affiliations:** 1 School of Public Health University College Cork Cork Ireland; 2 School of Applied Psychology University College Cork Cork Ireland; 3 Sexual Health Centre Cork Ireland; 4 Health Information Systems Research Centre Cork University Business School University College Cork Cork Ireland; 5 Student Health Department University College Cork Cork Ireland

**Keywords:** illicit drug use, student health services, web-based intervention, mobile phone, harm reduction

## Abstract

**Background:**

Digital interventions have been identified as a possible tool for reducing the harm caused by illicit drug use among students attending higher education (ie, college students). However, the success of interventions in this area has been hampered by a lack of user involvement and behavior change theory in their design. The My Understanding of Substance use Experiences (MyUSE) project combines a rigorous user-centered design (UCD) methodology and a robust behavioral change framework to develop a digitally delivered harm reduction intervention for illicit drug use among students in higher education.

**Objective:**

This project aims to design and develop a digital intervention that targets drug use–related harm among students in higher education.

**Methods:**

The MyUSE project will take place over 3 phases. The first phase was exploratory in nature, involving 3 systematic reviews, a large survey, and student workshops to gather a comprehensive evidence base to guide the project. The second phase is the development stage of the project, involving the use of the Behavior Change Wheel theoretical framework to determine the behavior change techniques of the intervention and the use of the UCD methodology to guide the development of the digital intervention. The third phase is the evaluation stage, whereby the intervention will undergo a 5-stage evaluation process to comprehensively evaluate its impacts.

**Results:**

The exploratory phase 1 of the MyUSE project was completed in December 2018. Phase 2 is currently underway, and phase 3 is due to begin in September 2020.

**Conclusions:**

Higher education institutions (HEIs) are ideally placed to intervene and support students in the area of illicit drug use but are constrained by limited resources. Current digital interventions in this area are sparse and have several weaknesses. The MyUSE project combines a UCD approach with a robust behavior change framework to develop a digitally delivered intervention that is economically viable, effective in changing behavior, usable and acceptable to students, and able to sustain long-term implementation in HEIs.

**International Registered Report Identifier (IRRID):**

DERR1-10.2196/17829

## Introduction

### Background

The use of illicit drugs among students in higher education is a growing public health issue, with the annual prevalence of illicit drug use among students increasing gradually over the past 10 years [1]. Approximately one-fourth of higher education students report current use of an illicit drug [1-4], placing them at high risk of experiencing a myriad of academic, social, physical, and mental harms [3,5-16], with the risks particularly high for first-year students [5,17,18]. In particular, a recent study in Ireland found that 50% of young adults present with at least a low level of problems resulting from drug use [19]. As a result, higher education institutions (HEIs), such as universities, institutions of technology, or colleges of higher education, are ideally placed to intervene to reduce harm from drug use among student populations. However, student support services are limited in their capacity to deliver face-to-face interventions to large student bodies [20]. Students may be unlikely to recognize a need for, or be reluctant to seek help or support [21,22]; thus, alternative delivery methods should be considered. Digital interventions have been developed that target a range of potentially harmful behaviors, including alcohol consumption [23-27], smoking [20,28], and illicit drug use [29-35]. Despite the initial optimism surrounding the effectiveness of such interventions, many have failed to achieve positive results [26,27,32-34]. Two of the potential reasons for this are a lack of user involvement in the development of such interventions and a lack of a theory-driven behavior change framework to inform their design and development.

The development of digital interventions should explore the needs of end users in their context [36]; involve those users throughout the process of designing, developing, and evaluating a new intervention; and use a systematic approach to synthesize the available evidence to select the most precise behavioral change components to maximize intervention outcomes [37]. The user-centered design (UCD) methodology is an iterative process that requires the early and active engagement of the target user through a number of activities including the development of user profiles and early prototyping and evaluation of the intervention [38]. The implementation of the UCD process is critical to ensuring user engagement with the intervention and subsequently enhancing the effectiveness of the behavior change techniques (BCTs) employed [39]. In addition, digital interventions are more likely to be effective if their active components employ relevant mechanisms of action, such as a theoretically informed understanding of the motivations for change in their target population [40]. Digital interventions focused on enhancing health behaviors, such as harm reduction practices or substance use cessation, highlight that user involvement plays a key role in achieving the objectives of the intervention [41-43]. At present, few of the existing digital harm reduction interventions describe the process involved in their content development and feature selection. This narrows the opportunity to replicate effective interventions, synthesize evidence based on theoretical premises, or understand the causal mechanisms that facilitate behavior change [44]. The intention of this project is to employ a UCD approach and to incorporate behavioral theory processes in the design and development of a digital intervention that targets drug use–related harm among students in higher education. Mummah et al [45] outlined the Integrate, Design, Assess, and Share (IDEAS) framework, a comprehensive 4-stage process to guide the development and evaluation of digital interventions, incorporating behavioral theory, design thinking, and evaluation. Utilizing the IDEAS framework, this project will incorporate the UCD methodology as a core research toolkit along with the Behavior Change Wheel (BCW) framework [46], embedded in an interdisciplinary design approach, to develop and evaluate a digital intervention. This study outlines the protocol for the development of a digital harm reduction intervention for illicit drug use specifically designed for students in higher education.

### The My Understanding of Substance Use Experiences Project

The Irish Government has published an 8-year strategy to address the harms caused by alcohol and drug use in Irish society. The strategy, “Reducing Harm, Supporting Recovery, a health led response to drug and alcohol use in Ireland 2017-2025,” identifies “the development of IT/web-based drug education, harm reduction and brief advice tools targeted at higher education students” as a key element of the prevention strategy. The strategy further states that “the engagement of people who use drugs and/or services in the development and roll-out of any awareness campaigns is particularly important to ensure relevance and accuracy” [47].

The increasing prevalence of drug use among higher education students in Ireland highlights the need for a theoretically robust, specifically developed program to support students to enjoy well-being and minimize the harms associated with drug use, using both prevention and intervention approaches. In response to this growing student health issue, University College Cork (UCC) established an interdisciplinary team and developed a proposal for the “My Understanding of Substance use Experiences” (MyUSE) project. The project aims to develop, implement, and evaluate a digitally delivered harm reduction intervention targeting both those who use illicit drugs and those who do not in the higher education setting. For the purpose of this project, we define illicit drug use as “the use of substances which have not been prescribed, with the exception of alcohol and tobacco, or the use of prescription medication not as prescribed.” This project is fully supported by a grant from UCC, Ireland, emphasizing the university’s commitment to the welfare of its students. This study presents the research phases, methods, and processes utilized in the MyUSE project.

## Methods

### Overview

**Figure 1:**
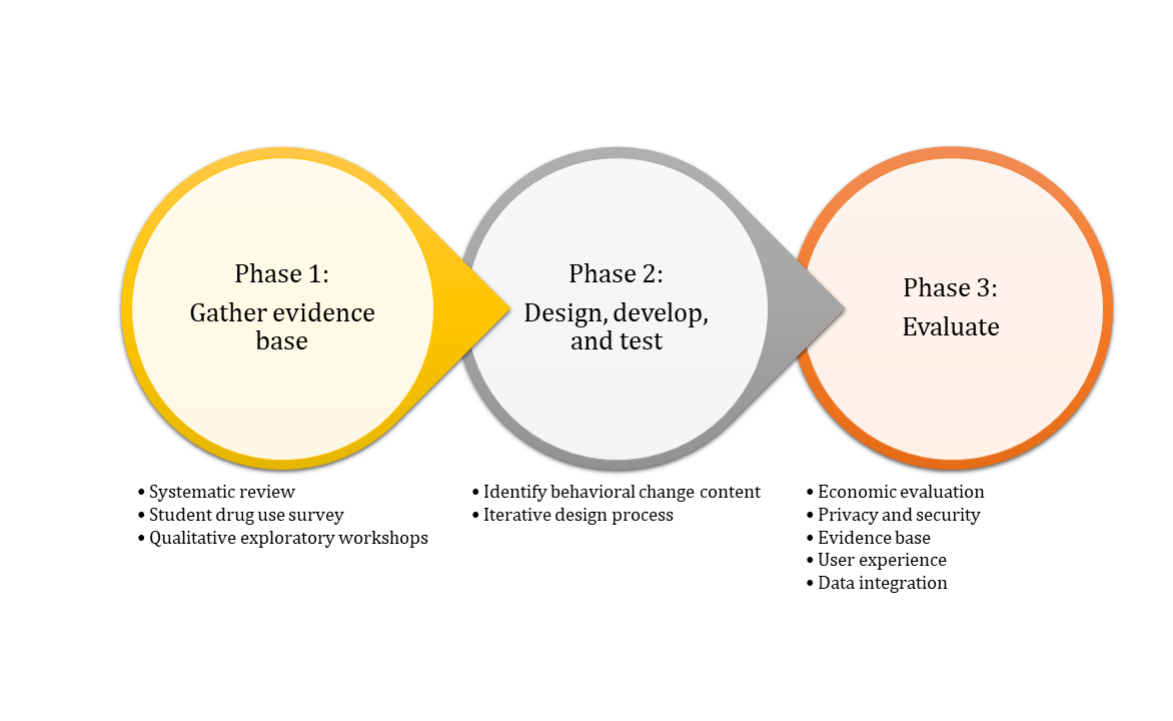
Objectives of the MyUSE (My Understanding of Substance use Experiences) project.

The MyUSE project will be undertaken over 36 months in 3 phases, combining UCD and BCW methodologies, guided by the IDEAS framework. The first phase focuses on establishing the evidence base around digital interventions for drug use, drug use and nonuse trends, and behaviors, through systematic literature reviews and surveys and gathering a deep understanding of the target population through qualitative focus groups. The second phase focuses on the design and development of the intervention, involving the identification of the behavioral change components, and the development of the digital intervention, involving the users at each phase as part of an iterative design process. The final phase will consist of a comprehensive, stepwise evaluation. Learnings from each phase of the project will be shared to inform future intervention development in this area. The project will contribute to the broader seminal body of research for both researchers and practitioners, including health care professionals, student health services, and software developers, by producing academic outputs and disseminating findings at national and international conferences to ensure that all results and learnings from this project are shared widely. This project will be developed in accordance with the 10 principles of the code of conduct for data-driven health and care technology, as outlined by the UK Department of Health and Social Care [48]. These principles were implemented to enable the development and adoption of safe, ethical, and effective data-driven health and care technologies and to incorporate principles such as understanding user needs and defining outcomes, transparency, and accountability. An overview of the 3 phases of the MyUSE project is outlined in Figure 1, along with the specific objectives for each phase.

### Project Objectives

The MyUSE project objectives are as follows:

1. To systematically review the relevant literature across 3 areas: (1) the use of UCD practices in similar interventions, (2) the effectiveness of similar interventions, and (3) the motivations for changing drug use in a higher education population (phase 1).
2. To design a survey instrument that will capture the drug use patterns of students and identify their capabilities, opportunities, and motivations for change (phase 1).
3. To conduct qualitative exploratory workshops with students to gain a deep understanding of the characteristics of end users and scenarios within which a student may decide to engage with an intervention of this nature (phase 1).
4. To develop intervention content by identifying the capabilities, opportunities, and motivations for changing (or in the case of nonusers, reinforcing) the targeted behaviors and by employing the BCW framework [46], which addresses the specific characteristics, needs, and behaviors of students in higher education (phase 2).
5. To develop the digital intervention through an iterative design, development, and test process, involving the end user (ie, students in higher education) in each stage of development and decision making (phase 2).
6. To conduct a comprehensive evaluation of the MyUSE intervention following a stepwise approach (phase 3).

### Phase 1: Gather the Evidence Base

The first phase of the MyUSE project was carried out over a 15-month period from October 2017 to December 2018. This phase included a systematic interrogation of the literature in the area of digital interventions and student drug use in higher education settings; qualitative exploratory workshops with higher education students to identify characteristics, needs, goals, and values of target users; and the design of a survey to assess baseline drug use behaviors and trends. At the outset of the project, a public and patient involvement group of student partners was assembled based on guidance from the National Institute for Health Research [49]. The *Student Advisory Group* (SAG) will inform and guide project development from the student perspective as the key stakeholders in the intervention.

The first recruitment of the SAG took place in January 2018. Academic leads on the MyUSE project provided information about the opportunity to join the SAG through class email lists. A total of 10 undergraduate students from 3 disciplines (Public Health, Applied Psychology, and Information Systems) signed up to the group. Members of the group are never asked to disclose their personal experiences with drug use. To date, 10 meetings have been held with the SAG. The meetings follow an informal discussion style format; members of the MyUSE team present a particular piece of work (eg, survey questions, design idea, etc); and the group engages in discussion around the student’s perception and areas of improvement. Brief notes are recorded throughout the meeting. The MyUSE team holds a debriefing session following each meeting to discuss key points arising from the meetings. A second round of recruitment took place in January 2020 as a number of SAG members had recently completed their studies or decided to leave the group.

#### Systematic Reviews

At the outset of the MyUSE project, 3 systematic reviews were conducted with the aim to identify, gather, synthesize, and analyze all relevant research to, first, assess the potential effectiveness of digital behavioral change interventions in this area and, second, to guide the project methodology.

The first review, “A systematic review of the effectiveness of digital interventions for illicit drug misuse harm” (n=8 studies, reported elsewhere [50]), was conducted to assess the effectiveness of digital interventions for drug use harm reduction in student populations. Modest success has been reported for alcohol and tobacco harm reduction interventions [23-25,28], but the differences between legal and illegal drug use with regard to the user’s related behavior may limit the extrapolation of those results to illicit drug users. Therefore, it was important to carry out a review specifically targeting digital interventions for illicit drug use to assess their overall effectiveness. The review reported modest positive outcomes for harm reduction in 5 of the 8 included studies. However, the overall quality of the included studies was weak, and few studies focused solely on illicit drug use (including smoking and/or alcohol use) and those that did focused only on marijuana [29-31]. In addition, there was very little information provided on the involvement of users in the design of the interventions included in this review.

The second review, “A systematic review of user-centered design practices in illicit drug use interventions for higher education students” (n=7 studies, reported elsewhere [51]), was conducted to investigate the previous interaction with UCD practices in the development of similar interventions to guide the development of the MyUSE intervention and our adoption of the UCD methodology. The review revealed that limited consideration had been given to the end user experience (UX), and there had been minimal engagement with UCD practices. Failure to engage users in the design and evaluation of digital interventions would have a significant influence on their effectiveness and sustainability in normal user conditions [39]. This review highlighted a gap in the current processes for intervention design in this area.

The third review, “Motivational factors related to higher education student’s decision to decrease or cease drug use: A scoping review” (n=3 studies, manuscript in preparation), was conducted to explore students’ motivations to change their drug use. A considerable amount of research has been conducted to explore the motivations for beginning or continuing drug use, but a scant number of studies have examined the motivations to reduce or stop drug use behaviors. This review reported that the sole identification of the adverse consequences of drug use is not sufficient to prompt students to change their current pattern of use. The findings also indicate that a motivation to reduce or stop drug use behavior may emerge from multiple cumulative and/or interactive factors [52], and the identification of consistent negative effects across several life domains may be necessary as a precedent for change. Findings from this review highlight how motives relating to the perceived social acceptability of various behaviors can facilitate behavior changes and how increasing awareness of individual decision making regarding drug use can also motivate changes in the use of illicit drugs.

#### Qualitative Exploratory Workshops

During this phase of the project, 8 exploratory workshops were conducted with 31 undergraduate students between December 2017 and February 2018. The workshops utilized a UCD methodology known as persona building. Persona building attempts to capture the user’s expectations, prior experiences, and anticipated behaviors, allowing developers to identify with and meaningfully communicate with the target user [53]. The workshops invited the participants to create detailed personas based on their own understanding of nonuse, moderate use, and heavy use of drugs and to identify conflicts between drug use behavior and students’ values and interests.

1. Understanding the service user: The participants were presented with fictional end users and asked to build a persona for each user based on (1) demographic information, (2) personality, (3) relationships, (4) interests, (5) behavioral patterns, (6) goals, (7) challenges, (8) annoyances, (9) fears, and (10) social routine. Participants developed personas for characters with no use, moderate use, or heavy use of drugs.
2. Motivation for service use: The participants were asked to describe how their persona’s relationship with drugs may interfere with various aspects of their life, including mental and physical health, relationships, and work or study.
3. Understanding service interaction scenarios: The participants were asked to write a short story about the personas they had developed, describing the series of events that led to their recognition of a need or concern.

These exploratory workshops assisted in identifying and characterizing the types of users who will engage with this intervention and how the intervention can be tailored toward their needs, values, and goals. Finally, a large mapping exercise was undertaken by the project team to synthesize the information from previous research. Participants identified 5 distinct drug use archetypes: (1) the social butterfly, (2) the high achiever, (3) the pleasure seeker, (4) the approval seeker, and (5) the health enthusiast. Full details of the workshops are presented elsewhere [54].

#### Drug Use Behavior Survey

The drug use behavior survey was developed by undertaking an iterative process over 12 months. The SAG was consulted on several aspects of the survey design, including its length, language style, mode of delivery, and the content of the questions. Survey questions were developed under 6 sections to collect information on (1) demographics, (2) student life, (3) drug use, (4) the decision-making process, (5) motivations for use, and (6) behavior change. Sections 1 to 3 of the survey assessed illicit drug use trends using items from a number of validated questionnaires, including the Core Alcohol and Drug Survey [55], the Alcohol Smoking and Substance Involvement Screening Test V3 [56], and the European School Survey Project on Alcohol and Other Drugs [57]. Sections 4 to 6 were constructed to assess students’ capabilities, opportunities, and motivations relevant to drug use behaviors. An overview of the survey content is included in Multimedia Appendix 1.

The survey was distributed via email to a randomly selected, representative sample of UCC students at the beginning of the 2018-2019 academic year. Proportional sample sizes were calculated from each year group of students (undergraduate years 1-5 and all postgraduates) to ensure that samples were representative of each year of study. The sampling framework was then utilized by the Information Technology Department to select and distribute the survey to a single mailing list of 3770 students.

The results of this survey will provide baseline information on drug use and nonuse trends among university students and assist in the process of identifying effective BCTs through a synthesis analysis, following the BCW framework. Following this, the survey will be optimized for delivery on an annual basis to facilitate longitudinal data collection on the drug use trends and behaviors among higher education students. The survey will be offered to other Irish HEIs in an effort to create a national data set that can be used to inform policies and practices within HEIs.

### Phase 2: Intervention Design, Development, and Testing

This phase of the project was carried out over an 18-month period, from January 2019 to June 2020 and included the systematic identification of the BCTs that will be implemented in the intervention and the iterative design and testing of the intervention with a small sample of higher education students.

#### Behavioral Content Development

The BCW is a theoretical framework for designing interventions, developed by synthesizing 19 existing behavior change theories [46]. It encapsulates the Capabilities, Opportunities, Motivations, and Behavior (COM-B) model, which states that for a behavior change to occur, individuals should change one or more components of physical or psychological capacity, social or physical opportunity, and automatic or reflective motivation [44]. The COM-B model is grounded by the Theoretical Domain Framework [58], a separate tool that includes a taxonomy of BCTs [58], which facilitates decision making from a pool of 93 different BCTs.

The BCW has been used in many digital interventions [59-63]. The BCW provides a framework to link intervention outcomes with the mechanisms of action, which enables an evaluation of the intervention and mechanism. The BCT taxonomy component of the BCW allows for the identification of the *active ingredients*, the observable, replicable, and irreducible components of the intervention. There are several applications of the BCW in the substance use domain: StopAdvisor, a smoking cessation program uses 33 BCTs from the taxonomy in its digital intervention, including “identifying reasons for not wanting to smoke” and “providing information on consequences of smoking” [61]. Similarly, Breaking Free Online, a computer-assisted therapy for substance use disorders, uses 6 BCTs, including the *framing*, *reframing,* and *goal setting* techniques [60]. To our knowledge, the BCW framework has not previously been applied to drug use in higher education populations.

Following the BCW framework, the evidence from the exploratory workshops, scoping review, and the survey will be synthesized to fully understand the motivations to change drug use behavior in higher education students. The evidence synthesis will determine which of the 9 intervention functions and 7 policy categories from the BCW will be considered as potential means by which the intervention can facilitate behavior change or behavioral reinforcement in the target population. Finally, the selected intervention functions and policies will guide the identification of the most relevant BCTs, which will then be translated into digital components.

#### Iterative Design Process

**Figure 2:**
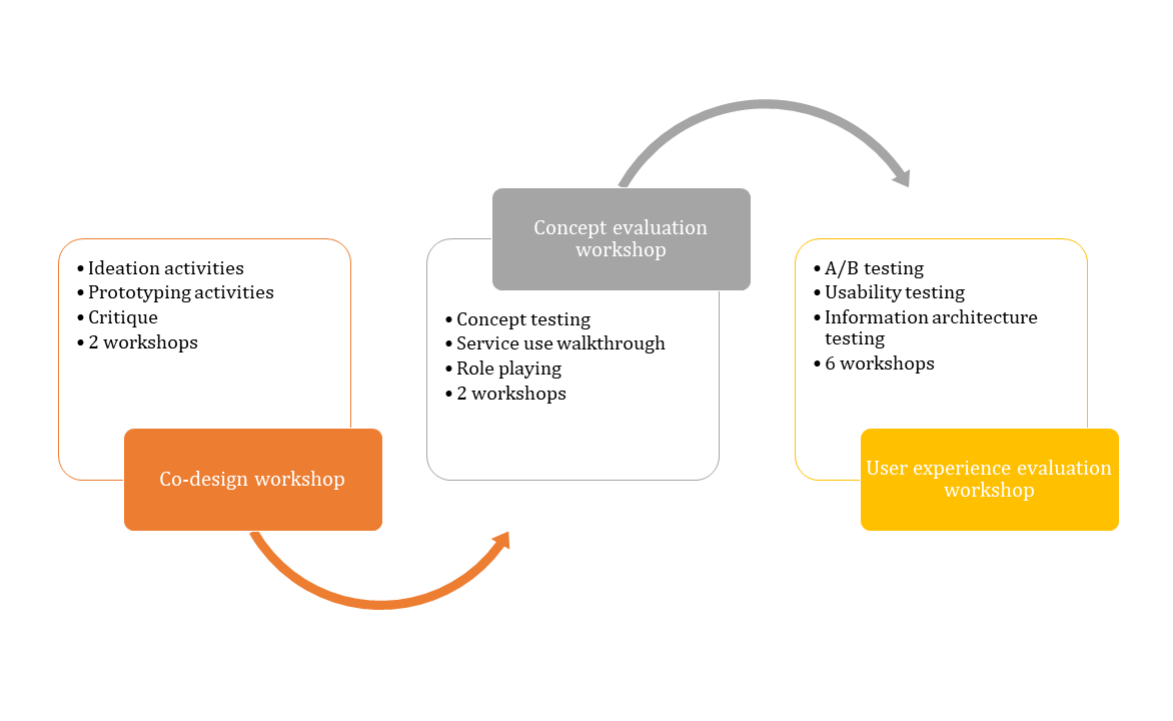
Iterative design process.

The iterative design process consists of 3 types of workshops: (1) an exploratory co-design workshop, (2) concept evaluation workshop, and (3) UX evaluation workshop, as illustrated in Figure 2. Recruitment for the workshops will follow a similar format to the phase 1 workshops. A call for participants will be periodically advertised through the Student’s Union social media platforms to create a *pool* of interested participants. Workshops will be advertised to the participant pool 2 weeks in advance. Participants will be able to participate in each type of workshop if they wish.

The co-design workshops will focus on the collaborative design of specific elements of the intervention. At this stage of the process, the project team would have determined (based on phase 1 evidence) which specific BCTs need to be included in the intervention. The co-design workshops are a process for deciding how to implement those specific BCTs in the intervention in a manner that is engaging and meaningful for participants. It is intended that at least two co-design workshops with groups of 6 to 8 participants each take place. The workshops consist of 3 steps: (1) ideation activities, (2) prototyping activities, and (3) critique.

1. Ideation activities: This involves a process similar to brainstorming, in which all stakeholders contribute their ideas to the various intervention BCTs. Participants will be presented with a specified activity that will be included in the intervention and will be asked to use flashcards, each containing a different element of delivery (eg, tone: funny, emotional, and trendy; mode: animated, pictures and text, and interactive visualization; and framing: mental health, well-being, and drug use, etc) to create a number of combinations of the delivery methods for the specified activity.
2. Prototyping activities: Having identified a number of ways in which each element of the intervention can be implemented, stakeholders will be asked to design low-fidelity prototypes, suggesting how these ideas could be implemented in the system, using paper, cards, pens, and post-its, to visualize their prototypes.
3. Critique: The facilitator will present a summary of the outcomes from the previous 2 exercises, and participants will engage in group discussion and critique of the ideas generated.

A small number of low-fidelity, digitalized intervention prototypes will be developed using Sketch prototyping software [64]. These will be based on the evidence gathered in phase 1 and the exploratory workshops and presented to students in a series of evaluative workshops. The concept evaluation workshops will consist of 3 parts: (1) concept testing, (2) service use walkthroughs, and (3) role playing.

1. Concept testing: During this workshop, a series of design components will be provided to the participants, supported by visual aids. Participants will be asked to provide feedback on these concepts [65].
2. Service use walkthroughs: Interface mockups will be used to guide participants through the task flow of the intervention. Participants will be asked to think aloud as they interact with each screen, verbalizing their actions, thoughts, and feelings as they attempt to achieve defined objectives. Participants will be asked to score the intervention across several criteria upon completion, such as functionality, ease of use, interactivity, clarity, and satisfaction [66].
3. Role playing: Using a UX analysis approach, participants will be presented with role scripts of a typical service use interaction and of the fictional personas developed in the exploratory workshops in phase 1. The scripts will describe a scene, plot motivations, and goals for each role. As participants act out the interaction scene, they will be asked to highlight areas where the experience with the intervention could be improved. The user will be asked to score the experience against defined criteria, and observers will also be asked to comment on the interaction scene [66].

It is expected that at least two concept evaluation workshops will take place with groups of 6 to 8 participants each before the project team reaches a decision on the final prototype to be employed for the MyUSE intervention to ensure that the final prototype adequately fulfills the needs of the student users. Each student workshop will be followed by a qualitative analysis of the students’ narratives and a half-day working meeting with the project team to discuss findings from the student workshops and incorporate changes accordingly.

The final task of the digital design and evaluation process will be iterative in nature, taking place over 12 months during which time the project team will work closely with students, allowing for further evaluation of the intervention to take place. Approximately 6 UX evaluation workshops will be held one-on-one with 3 to 5 students each, focusing on (1) A/B testing, (2) usability testing, and (3) information architecture testing The one-to-one nature of the final workshop will allow the facilitator to closely observe the participants as they interact with the intervention.

1. A/B testing: Pre-prepared sets of service interfaces will be provided to the participants, each representing 2 different design formats for task flow, interactive experience, or screen layout. The participants will be able to indicate which designs they prefer [65].
2. Usability testing: Participants will be provided with a general goal to achieve with the service. Using the think-aloud methodology, participants will be observed as they attempt to achieve the goal. Participants will be asked to assess the service in terms of its features and functionality, ease of use, navigation flow logic, and gestural design [67].
3. Information architecture testing: Participants will be asked to assess the ease of locating certain information and the intuitiveness of the structure of the information presented by the intervention. Participants will be provided with a series of cards representative of items in the navigation menu, and they will be asked to indicate which card they expect the information to be found under. Participants will be provided with cards that represent page headings and content sections. They will be asked to structure the pages and content in a manner that seems most logical to them [65].

### Phase 3: Evaluate and Disseminate

The final phase of the project will take place over 6 months, beginning in late 2020. The MyUSE project will follow a holistic approach to evaluation, following the stepwise framework proposed by Henson et al [68], modified for the needs of the MyUSE intervention. The framework comprises 5 ascending levels of evaluation, emphasizing the need to adequately assess the intervention at each level before proceeding to the next level. In keeping with the code of conduct for data-driven health and care technology [48], data security and privacy will form a core part of the intervention design. A secure, anonymous log-in feature will be developed, and a comprehensive data management plan will be established before the commencement of phase 3.

#### Level 1—Economic Evaluation

Reliable evidence of the economic effectiveness associated with eHealth remains to be limited [69]. We will undertake an analysis of the cost-effectiveness and budget impact associated with the MyUSE digital intervention. This will include understanding the benefits accrued by making this new student health intervention available, investigating value for money in the digital delivery model compared with traditional face-to-face interventions and establishing the quality of this digital health intervention. Furthermore, we need to develop an evidence base that will inform the business case for the potential delivery of MyUSE as an integral part of student health services in higher education.

#### Level 2—Privacy and Security

A Data Protection Impact Assessment will be carried out to assess the data protection risks associated with the MyUSE intervention. This will allow for the identification and mitigation of any data protection risks and assess the viability of the intervention [70]. Furthermore, the development of the intervention will implement the principles of *Privacy by Design* following a practical approach, with the inclusion of transparent, clear, and honest user agreements, terms and conditions, and consent process [71].

#### Level 3—Evidence Base

In September 2020, the finalized MyUSE intervention will be pilot tested in the UCC student population. A proportionally representative sample of UCC students will be invited to participate in the MyUSE pilot intervention during the registration period of semester 1, 2020-2021. A mixed methods approach will be used to evaluate the impact of the MyUSE intervention and to assess the UX. A pretest and posttest control group study will be conducted. Students will be randomly assigned to receive either the MyUSE intervention or an education-only control. This study will incorporate measures assessing the process of change variables, including the degree to which the intervention reduces the harm associated with drug use. The primary outcome measure will be the level of drug use problems, measured using the Drug Abuse Screening Test 10-item (DAST-10) questionnaire [72]. The DAST-10 is a brief screening tool suitable for self-administration that has been validated in college students [73].

The intervention and control groups will be assessed at 2 time points: T1 (semester 1), before the rollout of the MyUSE intervention, and T2 (semester 2), 3 months after the rollout of the MyUSE intervention. A sample of participants in the intervention (MyUSE) arm will be invited to focus groups to assess the UX and perceptions of the usefulness of the intervention at T2.

In addition, the baseline and subsequent cross-sectional survey of drug use trends collected in UCC (and other institutions in March 2020) will be used to map trends and will serve as an indicator of the long-term impacts of the MyUSE intervention within and across the student cohorts.

#### Level 4—Usability and Experience

The evaluation of usability and UX will begin during phase 2 of the project and will be achieved through a series of evaluative workshops. Following an agile approach, development in phase 2 will be conducted in 3 sprints, with the release of an updated prototype version at the end of each sprint. At least one evaluative workshop with 5 participants will take place following the release of each new version to inform further development. Furthermore, participants who receive the MyUSE intervention in the pilot evaluation will be invited to participate in focus groups and interviews to assess the usability and UX of the intervention.

#### Level 5—Data Integration

The final level of evaluation is complex and involves assessing the long-term clinical impact and sustainability of the intervention. A three-arm, clustered, controlled trial will be conducted with a number of institutions within Ireland to assess the behavioral impact and long-term scalability of MyUSE. All Irish HEIs will be invited to take part in the trial, and institutions will be allocated to receive the MyUSE intervention, an education-only control, or no intervention.

Primary outcome measures will assess the level of drug use risk (using the DAST-10 as outlined previously) and changes in targeted behaviors, such as decision making, behavioral awareness, and value progress using the Generalized Pliance Questionnaire [74], the Comprehensive assessment of Acceptance and Commitment Therapy processes questionnaire [75], and the Valuing Questionnaire [76]. Secondary outcome measures will assess user engagement using the analytics function built into the intervention (ie, number of clicks, time spent on each page, dropout point, etc). The trial will begin at the beginning of the academic year, with follow-ups after 3 months (end of semester 1) and again at 6 months (end of semester 2) to assess the longer-term impact of the intervention.

Interviews will be conducted with key stakeholders (ie, those responsible for the implementation, such as Student Health Department leads, Student Experience leads, and Student’s Union representatives) from institutions in the intervention arm to assess the barriers and facilitators of long-term implementation and scalability in the higher education setting.

### Dissemination

The MyUSE project will publish the results of the 3 systematic reviews, a process article describing the procedures of mapping harm reduction practices to the BCW, and findings from the iterative UCD workshops. The survey results and intervention evaluation will also be published. Furthermore, abstract and poster submissions will be shared at local, national, and international conferences to ensure that the findings and learnings from the MyUSE project are disseminated as widely as possible to contribute to the literature on intervention development. Furthermore, the MyUSE project will contribute to a much-needed national evidence base on the drug use trends and behaviors of higher education students. This will enable the development of evidence-based harm reduction policies and interventions at a national level.

## Results

Phase 1 of the MyUSE project was completed in October 2018, and phase 2 is currently underway. This project received funding for phase 1 in January 2017 and funding for phases 2 and 3 in May 2018. The project has received ethical approval from the Social Research Ethics Committee at UCC. Ethical approval for the student workshops was granted on November 17, 2017, and for the student survey on May 3, 2018.

In total, 3 systematic reviews were completed in phase 1. Two have been published [50,51]. A total of 8 persona building workshops with 31 students were conducted in phase 1. The findings from these workshops have been published elsewhere [54]. The student survey was distributed to 3770 UCC students in October 2018. The survey achieved a 30% response rate and a 20% completion rate.

## Discussion

### Summary

Students’ health and well-being services in HEIs are ideally placed to intervene and reduce the harm from drug use, yet they are limited in their capacity to reach large student populations [20]. In today’s increasingly connected society, digital devices provide the ideal platform to reach large student populations. However, previous digital interventions for illicit drug use in higher education students have seen only modest reductions in drug use–related harms [50], and many have suffered from problems with user engagement or lacked a strong theory-based framework as a foundation for BCTs [39]. Subsequently, there is little evidence to suggest that student populations had any role in the design, development, and evaluation of these interventions [51], despite the literature consistently identifying the importance of end user involvement [77-79].

There is currently very limited guidance available to research teams in the development of digital behavior change interventions [45]. The IDEAS framework [45] is one of the first to provide a systematic guide to intervention development, incorporating the essential components of behavioral theory, design thinking, and evaluation and dissemination. The MyUSE project aspires to avoid previous methodological caveats and aims to facilitate better exploration of the topic by adopting a mixed-method design, with the aim of maximizing the formation of a user-friendly, acceptable digital behavior change intervention. By applying UCD and BCW, formed under the umbrella of the IDEAS framework, we support the development of a context-driven design approach.

### Integrate

The first phase of IDEAS involves empathizing with target users, specifying the target behavior, and grounding in behavioral theory [46]. Our inclusion of a rigorous UCD process including persona building and user stories, in the design of this intervention, allows for a comprehensive understanding of the needs of the target population to be gained and will be used to inform the intervention design by identifying specific characteristics, needs, goals, and values of target users.

The use of the BCW framework allows us to specify the problem in behavioral terms (identify the specific components of drug use–related behaviors occurring within a higher education context) and to identify key sources of those behaviors (eg, habitual use of drugs among higher education students) that, when matched with specific intervention functions, can lead to the highly sensitive selection of BCTs holding the potential to maximize effective harm reduction practices. To the best of our knowledge, this is the first study in the area of drug use and experimentation within the university context that formulates the BCW as a primary framework for guiding the development of the content of a digital harm reduction intervention.

### Design

The second phase of the IDEAS framework involves ideating creative implementation strategies, prototyping potential products, gathering user feedback, and building a minimum viable product. The MyUSE intervention design will be undertaken in an iterative process, with several early prototypes introduced in succession to users for evaluation and feedback at each stage through evaluative workshops. The final design phase will take place over a 12-month period, again incorporating user feedback at each iteration. The inclusion of users throughout the intervention design, development, and evaluation will contribute to an intervention that is acceptable, user-friendly, and relatable to higher education students.

### Assess and Share

The final 2 phases of IDEAS include pilot testing, evaluation of efficacy, and sharing widely. The MyUSE intervention will be pilot tested with higher education students from the university before being rolled out on a larger scale. Finally, the systematic approach of researching and identifying effective components via the application of the BCW framework can be a useful working example for other researchers in the area and can contribute to the dearth of scientific knowledge on how implementation interventions are delivered in educational settings. The development of a survey that can be delivered on a national scale will provide a rationale for investment and opportunities for evidence-based policy and intervention development. Furthermore, the MyUSE intervention will be made available for HEIs across Ireland, with expansion to institutions in the United Kingdom and Europe a future possibility.

### Strengths and Limitations

This project combines 2 well-established and rigorous context-driven design methodologies in a systematic and transparent manner. Using UCD methodologies, this project aspires to circumvent barriers related to the use of technology, contributing to an optimal use of the digital intervention. Consequently, the MyUSE project maximizes the chances for intervention effectiveness. This project contributes to the advancement of digital intervention development, detailing the multidisciplinary approach in a manner that future research teams can draw on and replicate. Furthermore, this intervention provides a blueprint for intervention designers and software developers undertaking this type of project in the future.

This project has several limitations. The intervention focuses exclusively on students in higher education declaring use, past use, or nonuse of drugs. However, students with drug use–related disorders, as clinically defined in the Diagnostic and Statistical Manual of Mental Disorders [80], and individuals with comorbid mental health problems or other psychosocial and behavioral problems are not targeted, as they traditionally need a more intensive level of health care.

### Conclusions

To reach the overarching goal of delivering a web-based harm reduction intervention for illicit drug use in higher education student populations, this project combines a rigorous UCD methodology and a multiphase process-based BCW framework. Both procedures are employed to tailor the intervention to the unique needs of a higher education student population and to be attractive, usable, and acceptable to this population. Students attending higher education will be included as participants in exploratory and evaluative workshops as well as partners in our SAG, involved in decision making throughout the design, development, and evaluation. By incorporating all the involved stakeholders, the project ensures that the design is calibrated to the needs of the users, addressing a growing, yet unmet need of higher education health care policies to provide an evidence-based public health intervention targeting at-risk student populations [81].

This project has several research and public health implications. First, the digital intervention has the potential to protect students in higher education from the harm caused by drug use. Second, the agile digital delivery of the intervention will allow policy makers and health practitioners to communicate and promote effective behavior change practices via a wide range of channels (eg, web-based and smartphone-adapted technologies, social media campaigns, etc) and in different settings where drug use occurs (eg, music festivals, universities’ events, clubs, etc). Finally, given that students declaring drug use rarely visit traditional university health centers [21,22], this intervention has the capacity to deliver support to those students who may use illegal drugs to a degree that may cause them harm but are not motivated enough or lack the awareness required to seek help and support.

## Appendix

Multimedia Appendix 1 MyUSE drug use survey overview. My Understanding of Substance use Experiences.

## Abbreviations

BCT: behavior change technique
BCW: Behavior Change Wheel
COM-B: Capabilities, Opportunities, Motivations, and Behavior
DAST-10: Drug Abuse Screening Test 10-item
HEI: higher education institution
IDEAS: Integrate, Design, Assess, and Share
MyUSE: My Understanding of Substance use Experiences
SAG: Student Advisory Group
UCC: University College Cork
UCD: user-centered design
UX: user experience
