# Supplementary material for: A Digital Substance-Use Harm Reduction Intervention for Students in Higher Education (MyUSE): Protocol for Project Development
Source: JMIR Res Protoc. 2020 Aug 27;9(8):e17829. doi: 10.2196/17829 (PMC7484779; doi:10.2196/17829)
Supplement: Multimedia Appendix 1 [file resprot_v9i8e17829_app1.docx]

**MyUSE Drug Use Survey Overview**

| **Section** | **Title** | **Questions** | **Content** |
| --- | --- | --- | --- |
| 1 | Demographics | 8 | Age, gender, student status, college affiliation, part-time work, living arrangements. |
| 2 | Student life | 2 | Club/society membership, socialisation habits |
| 3 | Drug use | 25 | Perception of student drug use, lifetime use, past year use, past month use and frequency, reasons for use/non-use, declaration of substances, age of first use, timing of use, poly-drug use, change in use, effects of use, consequences of use, |
| 4 | Decision making process | 4 | Harm reduction practices used, use planning, sourcing of drugs, future use. |
| 5 | Motivation for use | 8 | Influence of others, alcohol substitute, change in feelings and behaviors, opportunities for change, |
| 6 | Behavior change | 8 | Concern from others, desire to change, consideration of change, capability of change, attempts to change, risk assessment, perception of intervention effects. |
